# Baseline measures of primary health care team functioning and overall primary health care performance at Du Noon Community Health Centre

**DOI:** 10.4102/phcfm.v10i1.1458

**Published:** 2018-09-05

**Authors:** Shapi Mukiapini, Graham Bresick, Abdul-Rauf Sayed, Cynthia Le Grange

**Affiliations:** 1 Faculty of Health Sciences, University of Cape Town, South Africa

## Abstract

**Background:**

Research consistently demonstrates the importance of effective team work for improving the quality of health care. We conducted a baseline measure of primary health care (PHC) team effectiveness and overall PHC performance at a primary care facility.

**Aim:**

To improve PHC team effectiveness and ultimately the quality and user experience of primary care at a community health centre (CHC).

**Setting:**

Du Noon CHC in the southern and western substructure of the Cape Town Metro district services (MDHS).

**Methods:**

A cross-sectional study using a combination of the Nominal Group Technique (NGT) consensus method and the South African Primary Care Assessment Tool (ZA PCAT) to assess PHC team effectiveness and PHC organisation and performance.

**Results:**

The ZA PCAT was administered to 110 CHC users (patients) and 12 providers (doctors and clinical nurse practitioners). Data from 20 PHC team members showed they perceived their team as well functioning (70% agreement on a 7-item PHC team assessment tool incorporated into the ZA PCAT). The NGT method achieved participant (20) consensus on communication and leadership as the main challenges to effective team functioning and on ideas to overcome the challenges. The ZA PCAT user data showed 18.2% of users rated first contact access as acceptable to good; 47.3% of users rated ongoing care as acceptable to good. Provider data showed that 33% of providers rated first contact access as acceptable to good; 25% of providers rated ongoing care as acceptable to good. First contact access received the lowest acceptable to good score (18.2%) and comprehensiveness (services available) the highest score (88.2%) from users. For the providers, the lowest acceptable to good score was for ongoing care (25%) and the highest acceptable to good score was for primary health care team availability (100%). The ZA PCAT total primary scores were good (above 60%) for both users and providers but moderately higher for the providers.

**Conclusion:**

Knowledge of how teams perceive their effectiveness can motivate them to generate ideas for improving performance. There were discrepancies between providers’ assessment of team functioning using the ZA PCAT measure and the NGT method results. The ZA PCAT also showed differences between providers’ and users’ perceptions of PHC performance – consistent with the findings of the multi-CHC Western Cape ZA PCAT study. These findings should encourage and support CHC and district level staff in their efforts to improve the quality and user experience of primary care, as well as PHC team performance.

## Introduction and background

Primary care is considered the backbone of the health system worldwide. In 1994, the Institute of Medicine (IOM) revised its first definition (1978) of primary care as the provision of integrated, accessible health care services by clinicians who are accountable for addressing a large majority of personal health care needs, developing a sustained partnership with patients and practising in the context of family and community.^1^

The performance of health systems has been a major concern of policy-makers for years. Over the past 25 years, many countries have introduced health sector reforms with the explicit aim of improving performance. There is now an extensive body of international and local literature on health reform. Current debates include how best to measure performance so that the impact of reforms can be assessed.^2,3^

Many studies in Africa have indicated the need for primary health care (PHC) reform, not merely to transform the health system but also to ensure a better life for all.^4^ Reforms include changes in financing, privatisation, decentralisation, integration of service delivery, and improvement of efficiency, equity and effectiveness of the health sector in general.^4^

The introduction of family medicine (FM) as a specialist clinical discipline in health care in Africa has highlighted the need for deeper reforms in the health system.^5^ At its fifth annual conference (2012) held at Victoria Falls, Zimbabwe, the Primafamed Network (a network of Academic FM departments in sub-Saharan Africa) participants from 20 countries agreed on PHC reforms in sub-Saharan Africa in line with the World Health Assembly resolutions. These included:

… to train and retrain adequate numbers of health workers with appropriate skill-mix, including primary care nurses, midwives, allied health professionals and family physicians, able to work in a multidisciplinary context, in cooperation with non-professional community health workers, in order to respond effectively to the people’s health needs.^6^

In South Africa, specific legislative and policy reforms in the health sector include the re-engineering of PHC with the necessary strengthening of the district health system, greater emphasis on the delivery of community-based services, a focus on social determinants of health, a renewed focus on quality assurance and improvement, and the implementation of a national health insurance (NHI) as a financing mechanism to promote universal coverage.^7^

Multidisciplinary teamwork has been advocated in numerous reports, policy documents and studies globally as a way to provide high-quality and efficient health and social care to populations.^8^ The best and most cost-effective outcomes for patients and clients are achieved when professionals work together to generate innovations that ensure progress in practice and service – as noted by Borill et al., in their report on the effectiveness of health care teams in the United Kingdom (UK) national health service.^8^

A well-functioning PHC team is essential for a patient-centred, coordinated and effective health care delivery system.^9^ Assessing PHC team functioning is therefore important for improving team effectiveness. Primary care has evolved over the years from a solo practitioner model where one practitioner provides patient care to a team model where more than one category of health worker is involved in patient care.^10^ With the advent of new technology and the availability of a wide range of information to health care providers and patients, not only has it become more difficult for one clinician to provide care in isolation but it is also potentially harmful.^10^

The concept of a health care team was initially implemented at the beginning of the 20th century to coordinate work. Teams are now an integral feature of health care delivery in primary care, as well as acute and long-term care settings.^11^ Cohen and Bailey defined ‘team’ as a collection of individuals who are interdependent in their tasks, who see themselves and who are seen by others as an intact social entity embedded in one or more large social systems, and who manage their relationship across organisational boundaries.^12^ Teams are also defined and classified according to the attributes such as task type, team duration, purpose, interdependence and autonomy.^12^ Studies have established the core principles and values of effective team functioning in primary care as well as health care in general. High-functioning teams are characterised by members who hold shared goals and shared knowledge, and who demonstrate high-quality communication that is timely, frequent, accurate and focused on problem-solving.^13^ Cromp et al. in their study on barriers and facilitators of team-based care identified that meeting with structured agendas promote high-quality communication, explicit standardised roles, clarified expectations and made roles more transparent to all members.^14^

Mitchell et al. in their work (2013) on core principles and values of effective team-based health care, identified five personal values that characterise the most effective members of high-functioning teams in health care: honesty, creativity, humility, curiosity and discipline. They also identified five principles that characterise a high-functioning team: shared goals, clear roles, mutual trust, effective communication and measurable processes and outcomes.^9^ In summary, effective communication, shared goals and good coordination appear to be the cornerstone of a high-functioning multidisciplinary care team. The composition of a team depends on the context; therefore, each team is unique but all teams aim to provide the best care for patients.

The implementation of the health care team in the early 20th century has increased the need to assess the functioning or effectiveness of health care teams.^11^ As more organisations implement team work, it is becoming increasingly important to measure team functioning (effectiveness). One reason for this is the likelihood that the more effectively a team functions, the more benefits they are likely to realise from the work team structure such as a well-coordinated primary care system.^15^ On measuring PHC team functioning, Sundstrom (1999) defined team functioning (effectiveness) as ‘the extent to which a work team meets the performance expectations of key counterparts – managers, customers and others – while continuing to meet members’ expectations of work with the team’.^15^

### Measuring team functioning

Instruments such as the care process self-evaluation tool (DCPSET), the practice team environment checklist (PEC), palliative care assessment tool (PACA), the organisational leadership assessment (OLA) and the team survey have been used by researchers worldwide to study team effectiveness according to the type of team being assessed. In the United Kingdom, a health care team effectiveness project was commissioned by the Department of Health to determine whether and how multidisciplinary team work contributes to quality, efficiency and innovation in health care in the NHS.^16^ Ellershaw, in a study on the effectiveness of a hospital palliative care team using the PACA, found that a hospital’s palliative care team is effective at improving symptom control, facilitating understanding of the diagnosis and prognosis and contributing to the appropriate placement of patients.^17^

Schraagen and colleagues used observers to directly observe team performance and to code the non-routine events in a study on assessing and improving team work in cardiac surgery. This method had certain limitations; capturing observational data, by necessity subjective and observer-dependent means that many events could be missed.^18^

Mash et al. in their South African study on managing organisational change and practice teams used a structured questionnaire to assess the effectiveness of two PHC teams in one primary care facility (PCF) in the Western Cape. Each team comprised two doctors and two nurse practitioners. The study found that the perception of team effectiveness differed between the two teams. Factors included differences in team resilience, leadership style and communication.^19^

Lurie, Schultz and Lamanna, after reviewing different tools used to assess team functioning, found that the tools available at that time were very resource intensive and thus could not be frequently administered. Instead, they adapted the validated 29-item PEC, demonstrating that five items were sufficient to yield reliable estimates of team effectiveness – that is, using a brief teamwork-assessment instrument – a reliable five-question survey, derived from the original, validated PEC.^20^

PHC re-engineering, a major health sector reform initiative underway in the South African public health sector,^7^ promotes the role of the PHC team in health care delivery to communities. The PHC team, therefore, has an important role to play in achieving the goals of PHC re-engineering; team effectiveness will be a key element. However, a review of the literature did not reveal any local or national studies assessing PHC team effectiveness, suggesting that PHC team functioning (effectiveness) has to date not been audited in South African public sector primary care.

The Western Cape PCAT study, which assessed primary care organisation and performance in the Western Cape province, South Africa (2013),^21^ used the locally adapted and cross-culturally validated the South African version (ZA PCAT) of the original PCAT expanded (*E*) version (vs. short [S] version) developed in the United States. The ZA PCAT validation method and process resulted in a new PCAT domain,^21^ PHC team availability, to measure PHC performance in South Africa. The baseline study found that the team availability domain scores were generally good in the 13 primary care facilities (PFCs) studied, but were of limited value as these scores only determined the presence or absence of key PHC team members.^22^ Such information can easily be obtained from the PCF managers or staff establishment records. The PHC team domain questions (items) do not assess team functioning – surely a more useful measure as argued by the authors of the ZA PCAT baseline study paper.^11^ A team functioning sub-domain was, therefore, added to the ZA PCAT by the ZA PCAT authors by inserting Lurie’s validated team functioning instrument to measure team effectiveness.^20^

### Local context

This study was conducted at Du Noon Community Health Centre (CHC), a PCF with flagship status in the Cape Town MDHS and Western Cape province. Du Noon CHC has within the past two years moved from two previous locations to a new PCF in MDHS and Western Cape province, while continuing to serve the same community. It has almost doubled its staff complement which now includes a specialist family physician (FP) who oversees clinical governance and expanded its services to include social work, occupational therapy (OT), physiotherapy, dentistry, nutrition (dietician), psychiatry, a psychologist and maternity care. The CHC contains the biggest and most modern trauma unit among Cape Town CHCs and the first time a CHC trauma unit works directly with a secondary hospital (New Somerset Hospital) through which the CHC doctors rotate to gain experience.

With its increased capacity, an average of 300 patients are seen daily at the new facility, 80% of whom need chronic disease care. Du Noon CHC serves a diverse community in terms of culture, race, nationality and income. Lower-income patients mostly reside in Du Noon and Joe Slovo informal settlements and on local farms, whereas patients in the middle- and higher-income range reside in areas such as Parklands and Tableview. All form part of the Du Noon CHC catchment area. The new facility is located in an industrial area and away from residential areas – a challenge for patients with regard to accessibility.

The overall purpose of the study is to improve the quality and user experience of PHC at Du Noon CHC. By using the ZA PCAT, it extends the Western Cape PCAT Study to Du Noon CHC. The added PHC team functioning domain described above extends the baseline measure to include PHC team functioning. Study objectives included auditing PHC team functioning, achieving consensus on the top five barriers to better team functioning and top five interventions to improve team functioning, obtaining a baseline measure of PHC organisation and performance using the ZA PCAT, and describing the demographic and socio-economic profiles of Du Noon PCF users.

## Methodology

This cross-sectional, descriptive study used two performance audit instruments and the Nominal Group Technique (NGT). Lurie’s validated five-question survey tool was used to assess team functioning,^20^ and the cross-culturally validated ZA PCAT^21^ was used to obtain baseline measures for PHC organisation and performance. The NGT method was applied to enable PHC team members to generate a list of factors affecting their team functioning and to obtain consensus to their main challenges.

The authors of the ZA PCAT adapted the five-question survey tool to suit the South African context by adding two questions from the original 25-question tool^20^ (Box 1). As in the main ZA PCAT study,^22^ all three PHC stakeholders were surveyed using the expanded versions of the PCAT – the ZA PCAT (facility manager expanded [FE]) for PCF managers; ZA PCAT (adult expanded [AE]) for PCF adult users; and ZA PCAT (provider expanded [PE]) for providers (doctors and clinical nurse practitioners). The ZA PCAT measures primary care performance on the following domains (Table 1).

**TABLE 1:** Adapted South African Primary Care Assessment Tool.

Domains	Sub-domains
First contact	-
Ongoing care	-
Coordination	• General
	• Information system
Comprehensiveness	• Services available
	• Services provided
Family-centred	-
Community-orientated care	-
Culturally competent care	-
Primary care team	• Availability
	• Effectiveness (new sub-domain)

BOX 1 Seven-question tool adapted from Lurie’s five-question instrument.

**Primary care team functioning items:**

- This team encourages everyone to share ideas.
- Leadership in this team creates an environment where things can be accomplished.
- People in this team have the information that they need to do their jobs well.
- When people in this team experience a problem, they make a serious effort to figure out what is really going on.
- Everyone in the team feels able to act on the team vision.
- Working in this team is stressful (original PEC item re-inserted by ZA PCAT study team).
- The team appears to let setbacks and problems stop its change effort (original PEC item re-inserted by ZA PCAT study team).

ZA PCAT, South African Primary Assessment Tool; PEC, practice environment checklist.

The PHC team effectiveness sub-domain applies only to managers and clinicians (primary care users are not team members) and, therefore, only in the FE and PE instruments. The method used to administer the PCAT followed that of the Western Cape ZA PCAT baseline audit study.^21,22^

### Study population, sampling and data collection

Users (patients) who had attended Du Noon CHC for at least three previous visits and were 18 years and older were eligible to be surveyed using the ZA PCAT AE. The user sample size calculation followed the 2013 Western Cape PCAT study^22^ (on primary care measures derived from a previous PCAT study [2011]) – using an estimated mean total primary care score of between two PCFs of 2.5 and 2.9, respectively, with a standard deviation of 0.8. The minimum sample size required per PCF was 85 (*α* = 0.05 and a power = 90%). (The total number of users interviewed in 13 PCFs in the original 2013 study was 1432; the PCF with the smallest and largest sample size was 97 and 123 users, respectively.) Regarding user selection, this study aimed to interview 21 users per day – 3 trained fieldworkers administering the ZA PCAT AE to an average of 7 users per interviewer per day for a period of 1 week (the 2013 study showed it to be a reasonable daily number to ensure good quality interviews given the resource constraints). An average of 300 users are seen daily at Du Noon CHC, 80% on an appointment basis. On each study day, user folders were selected systematically (every nth folder) from the booked and unscheduled user streams following the order of admission. Folders for users with appointments are usually retrieved on the previous day by a clerk (80%: approx. 240 folders) and placed in a dedicated room (club room). Users without appointments (20%: approx. 60 folders) are admitted via a separate admission process on the day where either a new folder is opened or an existing folder is retrieved and sent to different room (preparation room). Users were selected from these two rooms to include booked and unbooked users. Every 15th folder was systematically selected from among the booked users (240/15 = 16) and every 10th folder of unscheduled users (60/10 = 6) using the inclusion and exclusion criteria until the designated number was reached for the day. In cases where a user did not meet eligibility criteria or did not consent, the next file was selected and so on.

User interviews were conducted by fieldworkers with prior experience in public health surveys before being trained in a two-day training workshop using the ZA PCAT training manual adapted from the original US PCAT manual. (The two-day training included special attention to confidentiality, data collection and management, as well as general interpersonal communication skills and those that apply specifically to ZA PCAT data collection.) Training included roleplayed interviews with trained investigators followed by supervised practice interviews at Du Noon CHC prior to data collection. The lead researcher had previously been trained in a similar workshop. One fieldworker had considerable experience with ZA PCAT data collection, quality control and fieldworker training, having been involved in the 2011 and 2013 studies. All the fieldworkers were fluent in at least in two of the three major languages spoken in the Western Cape (English, isiXhosa and Afrikaans). The three fieldworkers were directly supervised by an experienced research assistant. (Every study day started with a brief meeting where the process for the day was explained and tasks were allocated to them. The interviewer approached the systematically selected user at the two designated rooms as described in the sampling method section [club room and preparation room] and the consenting user was taken to a pre-identified space in the CHC for the interview.) Every effort was made not to delay the user receiving his or her care at the clinic on the day. Data quality checks were performed by the supervisor after each interview before interviewers proceeded to the next interview.

All providers (doctors and clinical nurse practitioners) working as permanent staff were invited into the study (*N* = 12); interns, community service doctors and locum practitioners were excluded. The Du Noon facility manager and all full-time operational managers were invited to participate (one general facility manager and four Head of Departments [HODs]). The ZA PCAT PE and FE were administered to the consenting providers (clinicians) and managers, respectively – completed by agreement at a staff meeting with the investigators present to assist where clarification was required. Each participant completed the questionnaire individually without discussion between participants. The PHC team functioning sub-domain was, therefore, administered to 17 clinicians and managers. The investigators later decided to extend the PHC team effectiveness audit to other staff categories as well – pharmacists, social workers, physiotherapists, dieticians and clerks – to include the wider PHC team (1–2 representatives were selected from these categories). In this way, three participants were added to the PHC team effectiveness sample.

The NGT method, developed in 1968 by Delbecq and Van de Ven, is used to obtain expert panel consensus on a topic of importance^23^. Panellists generate and score their responses to obtain consensus on their combined top-ranked items. The 6-step NGT method was used to identify and obtain consensus among the Du Noon managers and clinicians (expert panel) on the main factors that in their view determine PHC team functioning at Du Noon CHC, and possible interventions to improve team functioning.

Twelve permanent staff members of Du Noon CHC were purposively selected to participate in the NGT to ensure all departments were represented: two doctors, two clinical nurse practitioners (CNPs), one professional nurse, two midwives, one pharmacist, one clerk, one physiotherapist, one social worker and one dietician. It has been established that 9–12 participants are a suitable number for an NGT group.^24^ The NGT was held at Du Noon CHC and conducted by the investigators; 90 min were allocated to the NGT group session to allow participants to return to their work stations immediately after. The following two questions were used to generate the panel’s responses:

- What are the main challenges to effective team functioning at Du Noon CHC?
- How can team effectiveness be strengthened or improved at Du Noon CHC?

At the start of the session, an overview of the study was presented, and the aim of the NGT and NGT process was briefly explained to the panel by the principal investigator (Step one), followed by the presentation of the questions (Step two). Each NGT question was dealt with separately. Participants were given time to think and generate items in response to question one (Step 3: silent phase). Twenty-four items were generated by participants and listed on a flipchart in the round robin phase (Step 4: item generation phase). Sixteen items were remained after clarification (Step 5: item clarification phase) – merging or grouping similar items. Twenty items comprised the clarified list. During the prioritisation phase (Step 6), each participant scrutinised the clarified list and ranked the top five items most important to him or her on their sheets without discussion – one being the most important and five being the least important. During the final voting phase (Step 7), each participant marked their ranked choices directly on the clarified flipchart list before the investigators summed the number of votes for each item to determine the top five items identified by the voting process. The results were visible to the whole group.

Because of time constraint, the second question could not be taken through the 7-step NGT process. Instead, it was modified and directed specifically at the top-ranked item (poor communication) as follows: ‘what can be done to improve communication at Du Noon CHC in order to strengthen and/or improve team effectiveness?’ All the participants responded enthusiastically; suggestions were recorded directly onto the flipchart and results presented followed by a brief discussion and participant feedback on the NGT process.

The ZA PCAT data analysis followed the method used in the 2013 Western Cape PCAT study so that the study results can be compared with the 2013 study findings.^22^ The method of item scoring, analysis and formulation of results follows the steps in the PCAT manuals for the three expanded versions ZA PCAT AE, PE and FE, respectively, obtained from the Johns Hopkins Primary Care Policy Center. Data are analysed separately for each version. The PCAT Likert-type responses and analysis are identical for the user, practitioner and manager questionnaires. Responses are scored on a 1‒4 scale, with 1 indicating ‘definitely not’, 2 indicating ‘probably not’, 3 indicating ‘probably’ and 4 indicating ‘definitely’. A fifth, ‘not sure or don’t remember’, response option is scored as 2 (except for the comprehensiveness domain where ‘not sure or don’t remember’ is scored as 0). The PCAT methodology calculates the score for each sub-domain by summing the scores of the items in that sub-domain (after reverse coding of items, where required by the data analysis method) divided by the number of items to produce a mean score.^22^

Data were entered into Epidata, cleaned and exported to Stata version 12.0 for statistical analysis. The internal consistency of the scores for users was examined using Cronbach’s alpha coefficient. The Shapiro-Wilk test indicated that the PCAT scores were not normally distributed; hence, as done in the original study, we constructed a binary variable. A score ≥ 3 is considered to be ‘acceptable to good performance’ and a score < 3 as ‘inadequate to poor performance’. For all analyses, a *p*-value of less than 0.05 and a 95% confidence interval that did not span unity were considered the thresholds of statistical significance. The new domain (PHC team functioning) uses the same PCAT Likert-type responses, but was administered only to practitioners (PE) and facility managers (FE) and therefore cannot be compared with users’ results. They were analysed separately and summarised on a different graphic. Because of the small number of managers, the ZA PCAT FE were not analysed separately.

During the NGT process for Question 1, all 16 items that remained after clarification were entered into the final voting stage. Items were rated 1–5 according to the level of priority given by each participant. To sum the votes for each item, we chose a numerical value for each as follows: 1 = 5, 2 = 4, 3 = 3, 4 = 1 and 5 = 1. The values for each item were summed to determine the top-ranked item followed by the item ranked second and so on until the item ranked fifth overall. As noted above, the second question was rephrased to address the top-ranked item identified as the main challenge to team effectiveness at Du Noon, that is, poor communication. Eleven solutions were generated and listed but not ranked.

## Results

### Primary health care team effectiveness

**FIGURE 1:**
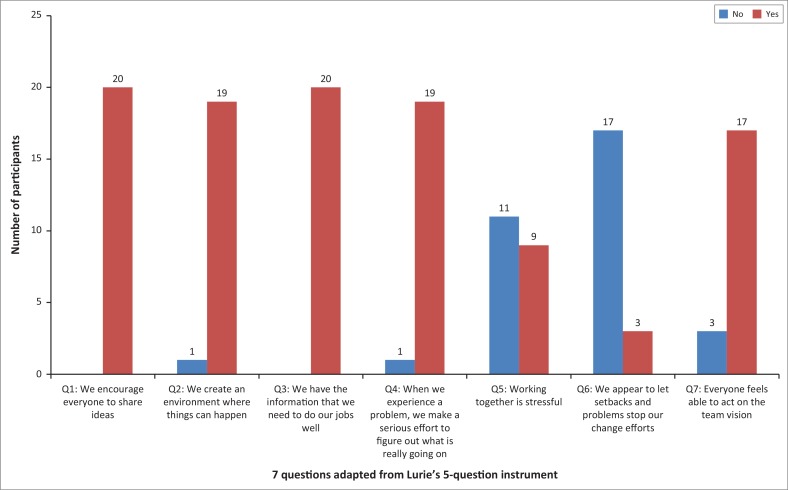
Proportion of providers in agreement or disagreement per item.

Adapted 7-question survey tool (PHC team functioning sub-domain): 20 providers completed this sub-domain section of ZA PCAT (12 clinicians and 8 providers from other CHC departments as described in the Method section). Figure 1 represents the number of providers who agreed or disagreed with each of the 7 items in the PHC team effectiveness domain; 45% of providers agreed with the statement (Q5) that working together is stressful versus 55% who disagreed. Working together is stressful (Q5) was the only item with high disparity among respondents. We encourage everyone to share ideas and (Q1) We have the information that we need to do our jobs well (Q3) both received 100% agreement. Over 80% of the providers agreed with the remaining items.

**FIGURE 2:**
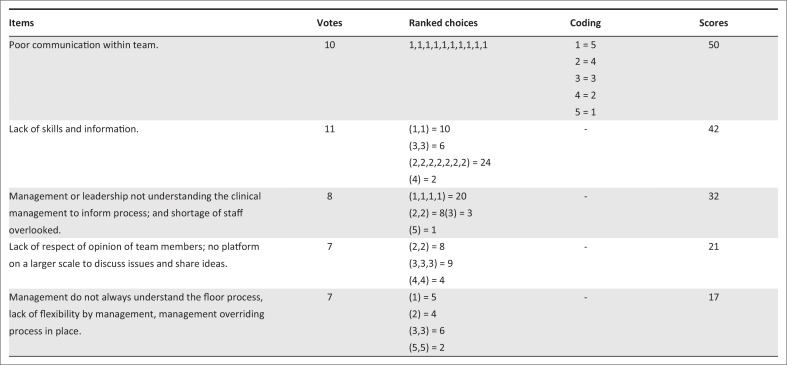
Final voting score of providers who participated in the Nominal Group Technique, in connection with question 1.

The NGT method: Figure 2 represents the final voting stage (Step 7) for NGT question 1 (What are the main challenges to effective team functioning at Du noon CHC?). Poor communication within the team emerged by consensus as the top challenge (total score of 50 points). During NGT Step 5 (clarification phase), items 3 and 5 should have been merged giving management–leadership 49 points – that is, regarded as an equally important challenge.

Box 2 lists the items generated in response to the NGT question 2 (What can be done to improve poor communication at Du Noon CHC in order to strengthen or improve team effectiveness at Du Noon CHC?). As noted above, because of time constraint, the NGT process for question 2 was stopped at the item clarification stage (Step 5) so that staff could return to their work stations. Steps 6 and 7 were, therefore, omitted.

BOX 2 Responses to nominal group technique question 2 (clarified list – Step 5).

**Responses:**

1. Talk directly to colleagues when referring a complex case (patient).
2. Change attitude of team member.
3. Standardise, put in writing communication around new process.
4. Engage team in changes, get team together to agree to the changes.
5. Respecting of team member’s opinion.
6. Inform all staff about changes timeously, effective use of notice board.
7. Management to undergo training in order to change their mind-set about team leader.
8. Team building.
9. Understand diversity in the team (cultural skills).
10. Meeting times: meeting to be scheduled when most people are free.
11. Meeting new staff regularly (buddy system).

### South African Primary Care assessment Tool results (excluding primary health care team effectiveness)

One hundred and ten users were interviewed using the ZA PCAT (AE) (acceptance rate 100%). All 110 questionnaires were analysed; 76% (84) of users were females and 23.6% (26) were males. Seventy (63.6%) patients were in the age range of 18–39 years; 29 (26.3%) patients were in the age range of 40–54 years; and 11 (10%) patients were in the age range of 55 years and over. The length of user association with the clinic was not longer than 17 months. All 12 permanently employed clinicians (doctors and clinical nurse practitioners) invited into the study completed the ZA PCAT (PE) (acceptance rate 100%). Of the permanently employed managers invited into the study (1 facility manager; 1 clinical manager and 3 HODs), 4 completed the ZA PCAT (FE) (acceptance rate 80%). As noted above, the managers’ data were not analysed separately because of the small sample.

**FIGURE 3:**
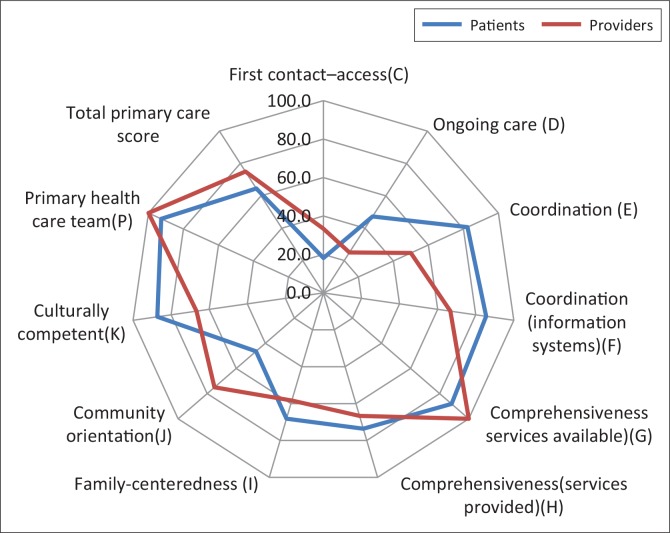
Radar graph summary of Table 2: Comparing percentage of patients and providers who rated performance as acceptable to good by domain.

Table 2 and Figure 3 summarise and compare the user and provider data by domain. The results were dichotomised (inadequate –poor, acceptable – good performance) following the method used in the 2013 study^22^; 18.2% of users rated first contact access as acceptable to good (i.e. where the primary care provider serves as the usual entry point into the health care system for each new health care need – except in the case of medical emergencies)^25^; 47.3% of users rated ongoing care as acceptable to good (i.e. the use of a regular source of care over time regardless of the presence or absence of disease or injury).^25^ The remaining sub-domains were rated as acceptable to good by at least 65% of the users; 33% of the providers (doctors and clinical nurse practitioners) rated performance on first contact access as acceptable to good; 25% rated ongoing care as acceptable to good; and the remaining sub-domains were rated as acceptable to good by at least 50% of providers.

**TABLE 2:** Age and gender distribution among users.

Demographic variable	*N*	%
**Gender**		
Male	26	23.6
Female	84	76.3
**Age (years)**		
18–39	70	63.6
40–55	29	26.3
55+	11	10.0

**TABLE 3:** Proportion of users and providers who rated performance as ‘acceptable to good’ by domain (i.e. scoring ≥ 3 on the primary care assessment tool Likert scale).

Sub-domains	Users (%)	Providers (%)
First contact access (C)	18.2	33.3
Ongoing care (D)	47.3	25.0
Coordination (E)	82.4	50.0
Coordination (information systems) (F)	85.5	66.7
Comprehensiveness (services available) (G)	88.2	100.0
Comprehensiveness (services provided) (H)	73.6	66.7
Family-centeredness (I)	68.2	58.3
Community orientation (J)	46.4	75.0
Culturally competent (K)	87.3	66.7
Primary health care team (available) (P)	92.7	100.0
Total primary care score	64.6	75.0

Among users, first contact access performed worst (18.2%) and comprehensiveness (services available) best (88.2%). Providers rated performance on ongoing care lowest (25%) and PHC team availability highest (100%). Total primary care performance was rated acceptable to good by users (64.6%) and providers (75%).

## Discussion

The user demographic profile (Table 2) indicates that Du Noon CHC serves largely female users (73.3%). This is consistent with other CHC-based studies in the Cape Town MDHS, including the Western Cape PCAT 2013 study.^22,26^ The smaller proportion (10%) of users aged ≥ 55 years (Table 2) reflects the fact that Du Noon and surrounding areas, being a more recently established community, have a much younger population than a long-established community such as Gugulethu where 40% of users attending the Gugulethu CHC were > 55 years of age,^26^ and explains the younger age range of the majority of users (63.6%) being 18–39 years. This can also be explained by the increased prevalence of chronic diseases such as hypertension and type 2 diabetes among younger people as well as the high prevalence of HIV and/or AIDS among young adults which in turn increases the number of visits per patient to the PCF. The HIV and/or AIDS prevalence of 14.50% among those aged 15–49 years in 2002 increased to 16.59% by 2015 in the same age group.^27^

### Primary health care team effectiveness

The PHC team effectiveness sub-domain – used for the first time in this study – was added to the ZA PCAT after the original 2013 study. Over 70% of respondents (CHC staff only) rated the PHC team at Du Noon CHC as effective (Cohen’s kappa, *k* ≥ 0.70). However, the NGT method identified poor communication and management–leadership as major factors determining team effectiveness at Du Noon CHC – an apparent discrepancy between the results of these two measures. The highest consensus rating given to poor communication is consistent with studies highlighting communication as a key factor in high-functioning PHC teams.^9,10,14,28^ It is possible that the discrepancy could be because of some providers having based their responses to the team effectiveness items in the ZA PCAT on their subteam’s functioning (e.g. trauma unit, dentistry and pharmacy), rather than overall multidisciplinary team functioning. Alexander et al. found that individuals who operate in more heterogeneous, larger teams have lower perceptions of team functioning.^29^

Effective communication within the PHC team is a key element necessary for integrated care.^30^ During NGT Step 8 (debriefing and discussion), staff emphasised the need for regular staff meetings where issues can be discussed and that these meetings should be scheduled at times when the majority of staff are able to attend. This is consistent with a UK study which showed that PHC team members spend relatively little time in team meetings and therefore have less opportunity to exchange information on individual patients across the disciplines.^30^ Recent studies of team functioning suggest that teams are most effective if all members actively engage in discussion to set team goals and methods. Good cross-disciplinary communication has a measurable, positive impact on the proper functioning of the PHC team.^30^ During Step 8, Du Noon staff also emphasised that respect for team members’ opinions, members’ attitudes, consideration of cultural diversity and introduction of new staff members to the whole team can potentially enhance communication within the team.

Leadership: as noted above, the NGT revealed that a well-functioning team needs a team leader and that all teams need support from leadership (management) to succeed.^31^ A systematic review by Gliggot highlighted effective leadership as a key factor for effective team work.^32^ Good leadership comprises multiple characteristics such as flexibility, recognition and appreciation of work done by the team; and knowledge of conditions that encourage effective functioning in different types of teams in particular settings.^31^ Knowledge of patient flow through the CHC (from the entrance of the building to consulting room), some knowledge on management of certain medical conditions and flexibility are some of the characteristics of good leadership that were emphasised as necessary in this context by the CHC staff (Figure 2 items 3 and 5). These characteristics are likely to promote strong leadership and better team effectiveness, resulting in better health outcomes in Du Noon. These characteristics are in line with findings in the literature regarding team leadership.^31,32,33^ Howard reported that leadership in PHC contributes to team work by unifying differences in a team and providing support for innovation.^33^ Taplin et al. recommended that the team leader should help teams map their work and clarify roles to improve functioning.^31^ This supports the need for attention to the floor (i.e. admission) process identified by Du Noon CHC providers. A team leader should positively influence the culture, composition and size of his or her team – all of which positively affect team outcomes. A team leader should also involve team members in decisions that affect the team, which in turn improves loyalty, cooperation and retention.^31^

Discussion during item clarification revealed that the facility manager’s roles and responsibilities are at times in conflict with those of clinicians, for example, the manager may prefer to admit all the patients who present at the CHC for health care on the day regardless of staff shortages, whereas providers will be more concerned about admissions impacting their clinical workload. Another factor raised was that managers frequently move staff from one post – for example, from preparation room to the tuberculosis (TB) room – to palliate a shortage of staff in one area irrespective of workload in another. Although CHC managers have direct responsibilities to district and provincial managers and the user community, as leaders they should also consider providers’ concerns when making decisions, for example, by using a locum service to reduce the impact of a staff shortage. Although at Du Noon CHC staff perceived their team as well functioning in the ZA PCAT audit, the NGT process revealed that they feel strongly that improved communication and a leadership mind-set shift are needed to improve team effectiveness.

### Performance on primary care domains

Overall primary care performance (total primary care score) was rated acceptable to good by 64% of users and 75% of providers (doctors and CNPs). Providers (and managers) assess the CHC’s performance from their perspectives – perspectives determined by their professional knowledge and the practice of primary care including any constraints in the provision of services, whereas users’ assessments of performance are largely determined by their actual experiences of primary care over the duration of their association with the CHC as their usual place of care. These very different but necessary perspectives may explain the discrepancy between user and provider performance scores. In addition, providers (and managers) are essentially commenting on their own professional performances, relative to the user experience of care, and may, therefore, err on the side of optimism – one reason the user experience is increasingly being recognised as an important element in evaluating and improving care. Research that deepens understanding of the differences between provider and user assessments of performance may help identify and implement interventions to improve PHC.

First contact access and ongoing care were scored acceptable to good by < 50% of users and providers. First contact access refers to primary care (rather than a higher level of care), being the usual entry point into the health system for each new health care need, other than for emergencies.^25^ During the report-back of the results, Du Noon staff suggested that the low rating could be attributed to staff shortages (e.g. clinicians) resulting in some patients not being seen on the day they present but given an appointment to attend on a later date instead, that is, limited access. Ongoing care includes continuity of care (relational) as well as the use of a regular source of care over time regardless of the presence or absence of disease or injury.^25^ It serves to build a long-term relationship between user and provider to enhance mutual trust. Less than 50% of users and providers (47% and 25%, respectively) rated this sub-domain as acceptable to good, compared to the Western Cape ZA PCAT study (2013) where over 50% of users and providers rated the domain acceptable to good. Our finding reflects more closely the findings of the two unpublished audits of continuity of care in Cape Town CHCs noted in the Western Cape study.^22^ These studies reported poor continuity of care where continuity was defined as users of chronic disease care seeing the same clinician for at least 66% of the consultations over a 2-year period. Our finding of 25% (providers) approximates the finding of 21.4% in one of the studies using a record audit of chronic disease patients. During the report-back of the study findings at Du Noon CHC, staff felt that operational factors, for example, how patients are allocated to clinicians, make it difficult for a patient to be seen by the same clinician at consecutive visits. Although Du Noon users have the opportunity to make regular use of the CHC for all their primary care needs, staff shortages – a challenge in many local CHCs – may be the biggest factor driving poor continuity of care which is known to result in fragmented care and poor outcomes.^34^

Community-orientated primary care (COPC) refers to care that seeks to understand and respond appropriately to the context and health needs of the community served. It includes involving community leaders and organisations and gathering information to determine and deliver the services that community needs. Less than 50% of patients rated COPC acceptable to good, whereas 75% of providers rated it acceptable to good – a sizable difference. During the report-back meeting, clinicians suggested that the low user score could be because of inadequate information regarding the services available to the community, for example, home-based care for TB and HIV and/or AIDS treatment. Access to information regarding services available in the community could be improved through the health committee and by the CHC health promoter, for example, using regular announcements and posters in waiting areas.

The remaining domains and sub-domains (excluding PHC team effectiveness not assessed by users), that is, coordination of care, comprehensiveness, family-centeredness, cultural competence and PHC team (availability), were scored acceptable to good by more than 60% of users and providers. Coordination of care refers to availability of information about previous health care and services used and the recognition that such information is important for ongoing care.^25^ Family-centred care recognises the family as a key participant in patient assessment and care.^35^ Research on families and health demonstrate the powerful influence of the family on health, illness and healing and the benefits of family-based interventions.^35^ Culturally competent care refers to care that respects the beliefs, interpersonal styles, attitudes and behaviours of patients in the context of their families and communities.^35^

### Limitations and strengths

Users were sampled during one week and may, therefore, not represent the user experience during other weeks of the year given changing operational and seasonal effects. However, the findings are similar to those of the Western Cape ZA PCAT study which used the same method and spread data collection across 13 CHCs over a number of months – suggesting that any impact of such effects may be minimal. Respondents’ assessments were based on their experience of primary care at Du Noon over the duration of their association with the CHC; inaccurate or incomplete recall of past experiences can affect responses. The number of managers at Du Noon CHC did not permit an adequate manager sample size; ZA PCAT FE data were therefore not analysed separately. The time constraint during the NGT session did not allow the second question eliciting ideas to improve team functioning to be taken through all the NGT stages. Useful insights on the team’s functioning were nevertheless obtained as a first step to improving performance.

The NGT method’s ability to create an equal opportunity for each team member to reflect and comment on the importance of team functioning for team effectiveness may have been the more important benefit of the exercise. Enabling team members themselves to determine and achieve consensus on the main items influencing team functioning, can be seen as a strength of this study. Team members were able to generate responses (individually) to improve team effectiveness based on their own experience and observations of their work environment and team climate. Although the structured NGT method necessarily limits discussion, the content of the discussion during item clarification phase (Step 5) and after the NGT session was completed (optional) indicated that the ZA PCAT audit of team effectiveness did not convey the full picture. Although the apparent discrepancy between the ZA PCAT measure of team effectiveness and the NGT results could be attributed to misunderstanding the team effectiveness sub-domain questions in the PCAT, the findings nevertheless highlight the value of applying more than one method of data collection to obtain a more granular understanding of the topic. The results have the potential to improve effectiveness if jointly implemented by providers and managers.

## Conclusions and recommendations

The findings of this study add to the existing 2013 Western Cape study database. Performance on the key features (domains) of primary care measured was similar to that found in the larger Western Cape study. Although the total primary care scores for users and providers were 64.6% and 75%, respectively, performance on individual domains show that there is room for improving the user experience of primary care and ultimately health outcomes. The contrast between user and provider perceptions of PHC performance and the poor performance rating given to first contact access by both users and providers are important findings for the attention of CHC and district managers.

This study employed a novel method to measure PHC team effectiveness in the Cape Town Metro District Health Services by incorporating a validated tool into the existing ZA PCAT. The results describe how a CHC-based PHC team perceives its effectiveness and how the NGT enabled the team to generate and reach consensus on items they believe affect their team functioning and effectiveness, as well as generate their own list of interventions to address these. Communication and leadership emerged as the main challenges to team effectiveness. They suggest a need for team leaders and managers to be more aware of their role in shaping teams and a need to focus on leadership in manager training that rewards team performance. It is hoped that the findings and NGT method experience will encourage and support efforts to improve team functioning at Du Noon CHC. As suggested by a Du Noon staff member during the report-back meeting, we recommend that NGT sessions be held on issues raised to generate and obtain consensus on interventions to improve performance.

The study findings do not determine specific interventions to improve team functioning and overall primary care performance. The authors believe nevertheless that they can guide Du Noon CHC managers and staff to improve primary care performance and organisation and improve users’ experience of care and should be considered at similar CHCs in Cape Town, the Western Cape province and elsewhere.
